# The Amsterdam Criteria and beyond: development of a Standardized Structured Reporting (SSR) tool for diagnosis of placental pathology

**DOI:** 10.1007/s00428-025-04143-0

**Published:** 2025-06-18

**Authors:** Ekaterina Bazyleva, Lotte E. van der Meeren, Mirthe H. Schoots, Linda M. Ernst, T. Yee Khong, Neil J. Sebire, Paul Seegers, Sanne J. Gordijn

**Affiliations:** 1 Belgian Society of Pathology, Brussels, Belgium; 2 Faculty of Medicine and Health Sciences, Ghent University, Ghent, Belgium; 3 Department of Obstetrics and Gynaecology, University Medical Center Groningen, University of Groningen, Hanzeplein 1, 9713 GZ Groningen, The Netherlands; 4 Department of Pathology, Leiden University Medical Center, University of Leiden, Leiden, The Netherlands; 5 Department of Pathology, Erasmus Medical Center, Rotterdam, The Netherlands; 6 Department of Pathology and Medical Biology, University Medical Center Groningen, University of Groningen, Hanzeplein 1, 9713 GZ Groningen, The Netherlands; 7 Department of Pathology and Laboratory Medicine, Evanston, IL, USA and Department of Pathology, Evanston Hospital, University of Chicago Pritzker School of Medicine, Endeavor Health, Chicago, IL USA; 8 Department of Anatomical Pathology, Women’s and Children’s Hospital, North Adelaide, South Australia; 9 Department of Pediatric Pathology, NIHR Great Ormond Street Hospital Biomedical Research Centre, London, UK; 10 Palga, National Pathology databank, Houten, Netherlands

**Keywords:** Placenta, Standardized Structured Reporting (SSR), Placental pathology, Amsterdam Criteria, Freedman-Ernst Placental Phenotype Classification, Diagnostic Accuracy, Histopathological Examination, Automated Calculations, Placental Lesions, Macroscopic and Microscopic Assessment, Data Standardization, Consensus guidelines, Research, Database Integration

## Abstract

The lack of standardized reporting in placental pathology limits research scalability and clinical application despite consensus-based criteria like the Amsterdam Criteria. This study presents the development and implementation of a comprehensive Standardized Structured Reporting (SSR) tool for placental pathology. Utilizing the LogicNets© platform, the tool incorporates the Amsterdam Criteria, Freedman placental phenotype classification system, and consensus recommendations on placental examination. Development involved collaboration with placental pathology experts, literature review, and iterative feedback from pathologists to address reporting inconsistencies and ensure practicality. The SSR tool integrates evidence-based standards for examining and phenotyping the placenta, including structured gross and microscopic analyses. It introduces control mechanisms for severity grading and phenotype assignment, enabling precise assessment of placental lesions, even in multiple births. Organized into sections—Demographics, Macroscopy, Microscopy, and Group and Phenotype—it efficiently records and analyzes data. Additional functionalities include automated calculations of gross placental features, built-in controls, and versatile documentation of microscopic observations. The tool is designed for seamless integration into clinical workflows. Implementing the SSR tool could enhance placental pathology reporting quality, completeness, and consistency, facilitating large-scale analyses. Discrete data capture enables robust research, potentially improving the clinical utility and understanding of placenta-mediated diseases. However, further validation is required before widespread adoption. Embracing SSR could standardize placental examination, improve clinical interpretation, and advance research in placental pathology for enhanced utility in both research and clinical care.

## Introduction

The placenta plays an essential role during pregnancy, serving as a temporary but vital organ with multiple functions, including delivering nutrition and oxygen to the developing fetus and supporting its maturation [1]. Suboptimal placental development and function can result in severe pregnancy complications such as preeclampsia, fetal growth restriction, preterm birth, and stillbirth, which are significant contributors to maternal and perinatal mortality and morbidity worldwide [2–6]. Despite the placenta’s critical role in these adverse outcomes, the frequency of formal placental histological examination post-delivery is notably low [7]. This underutilization is attributed to perceived issues with clinical utility, financial constraints in many healthcare systems, and a lack of standardized global pathology practices.

A major challenge in the field is the absence of uniform terminology and diagnostic criteria for placental lesions, which hampers the comparability of research studies and diminishes the clinical utility of their findings [8, 9]. The inconsistency in terminology, diagnostic criteria, and reporting methods across different pathologists and studies complicates the interpretation of placental pathology findings, thereby obstructing advancements in understanding and managing adverse pregnancy outcomes [10–12]. Standardizing placental pathological findings is a foundational step toward enhancing the tailoring of treatments in randomized controlled trials by identifying specific pathologies associated with subtypes of conditions, such as fetal growth restriction, which require differentiated therapeutic approaches [13, 14].

The establishment of comprehensive guidelines by the Society for Pediatric Pathology Perinatal Section from 2013 and the 2016 Amsterdam Placental Workshop Group Consensus Statement have provided a solid foundation for placental assessment [8, 15–19]. Notably, during the Dublin Consensus Meeting in 2019 further refined placental diagnostic criteria. These guidelines have been further enhanced by incorporating the Freedman-Ernst placental phenotype classification system, which is Amsterdam criteria-based and adds lesion severity and multiplicity to the diagnostic process, providing severity stratification based on placental phenotypes [20]. Despite the widespread acceptance of these consensus guidelines for placental examination established by expert panels, the majority of reports continue to use narrative (free text) formats [9, 15]. This approach stands in contrast to the structured and standardized synoptic reporting methods adopted by other pathology sub-disciplines, potentially limiting the utility of placental pathology reports in clinical practice.

Recent developments in web-based applications, including one supported by the Human Placenta Project and another developed by a member of the current group, represent significant advancements in the real-time classification of placental pathologies [21]. These applications facilitate the recording of detailed findings and the generation of comprehensive reports that include details on phenotype, pathology grades, and significant findings. They also offer commentary on phenotype prevalence and their clinical associations and allow for a narrative microscopic description, which can be customized for specific reports. Although they cover the most prevalent pathologies, they have not described a macroscopic examination approach to create a full pathology report. Their design does not contain automated calculations, and, most importantly, they do not contain a supporting database that safely captures data of cohorts. Despite these challenges, the application remains a valuable tool for phenotype identification, offering distinct advantages for its intended purpose.

In light of these additional needs for optimal standardized placenta reporting, we aimed to develop an SSR tool that addresses these challenges and sets a standard for complete placental pathology report to be submitted to a safe data repository. The tool should be suitable for clinical practice when a (automatically generated) conclusion is added and research as well as for both general pathologists in clinical practice as well as perinatal pathologists. With the development of the SSR tool for placenta pathology based on the Amsterdam Criteria and subclassifications, we advocate for changes in placental pathology practice to fully leverage the diagnostic potential of the placenta, thereby advancing perinatal care and research.

## Methods

The SSR tool was developed through a structured, sequential process to address the existing inconsistencies and completeness in reporting and enhance the quality of placental pathology examinations. The initial step involved assembling a multidisciplinary core team of experts in placental pathology known for their contributions to the Amsterdam Criteria and development of reporting tool and their clinical expertise as perinatal pathologists (SG and pathologists YK, LE, NS, MS, LM). This group was tasked with identifying the current challenges in reporting practices and defining the primary objectives for the SSR tool. The formation of this team established a foundation for the project, ensuring that the development of the tool was guided by comprehensive expertise and a clear understanding of the field’s needs and common practices.

The next phase focused on mapping the placental report protocol. This involved discussions regarding lesion classification, standardized terminology, and incorporation of data points critical for clinical assessment. The core of the SSR tool was informed by a literature review and consensus guidelines such as the Amsterdam Criteria and the Freedman-Ernst Placental Phenotype Classification System, ensuring the approach was rooted in evidence-based practice [8, 19–21]. Through collaborative online meetings and email exchanges, the team drafted an initial map of the placental report, laying out the structure and key components of the SSR tool development and future application.

With the protocol map as a guide, the Functional Design (FD) was developed in Microsoft Excel (version 16.77.1) and organized into several tabs corresponding to different aspects of placental assessment and anatomical location. The FD included internal field names, variables, conditions, and checks designed to support intelligent functionalities such as calculations and phenotype classification pathways. It featured a blend of core and non-core questions (to allow variation in the level of detail), various input methods, and educational content, all designed to ensure the capture of accurate and standardized data. After thorough revision, the multidisciplinary core team conducted a first-phase validation.

Once the FD was finalized, the development phase transitioned to building the SSR tool within the LogicNets© platform. This platform was chosen for its ability to accommodate the SSR tool’s complex requirements, calculation automation, comprehensive and secure database integration, and user-friendly interface for pathologists. The tool’s development in LogicNets© was a critical step in transforming the conceptual design into an application for daily practice.

### LogicNets© platform and supporting functions

**Fig. 1:**
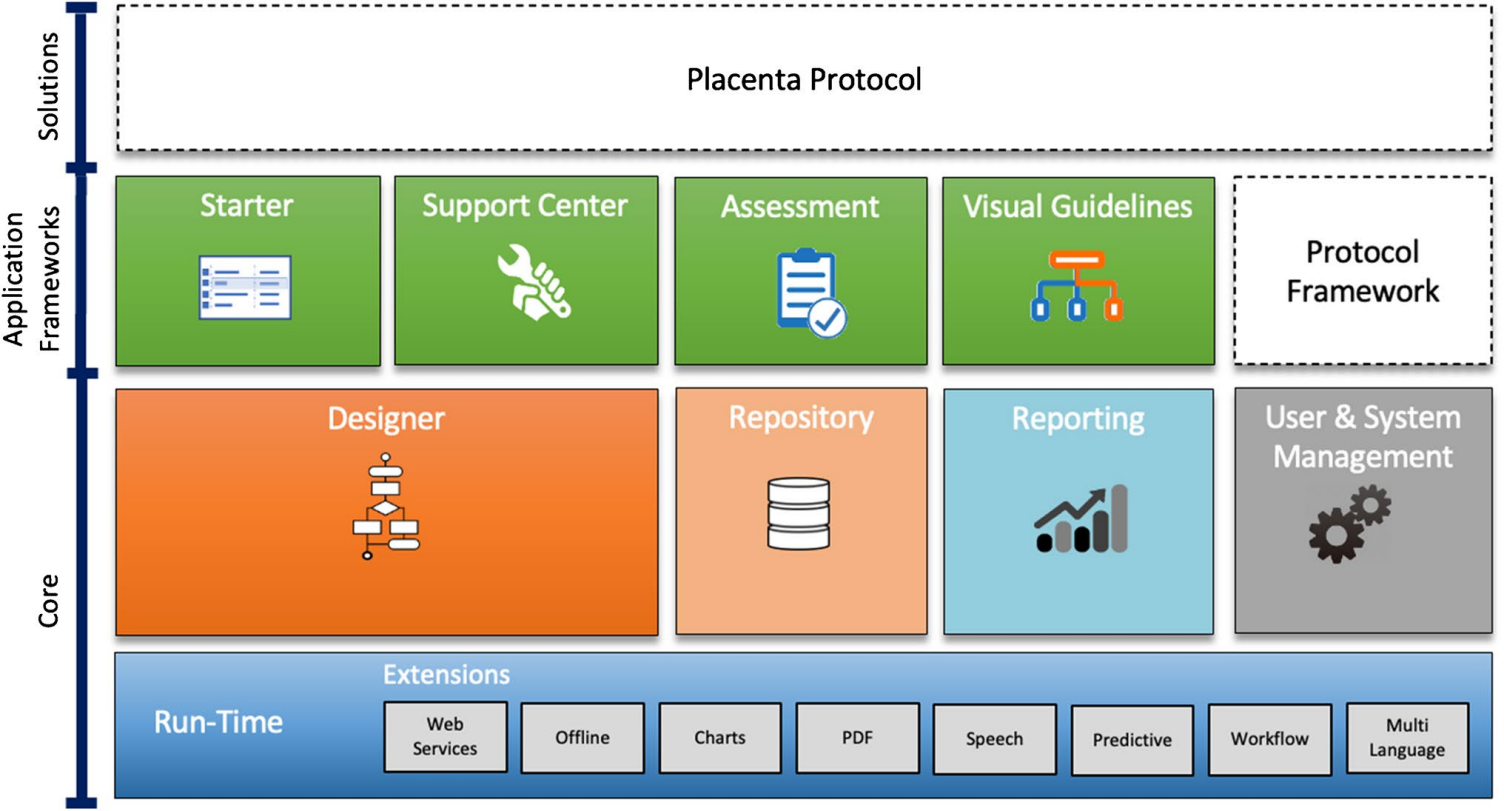
LogicNets application stack

The SSR tool was developed on the LogicNets© platform, incorporating various core and framework applications such as the Assessment Framework, the Starter, and supporting systems. Specifically, for the SSR tool, a Protocol Framework and the Placenta Protocol were developed (Figure 1).

### Validation

The validation process for the SSR tool involved an in-depth evaluation by the pathologists of the core team. They meticulously interacted with the tool’s interface to assess its usability and effectiveness in ensuring reliable and complete data capture. This critical phase aimed to ensure that the SSR tool met all the requirements for ease of use and accuracy in reporting essential pathology data, aligning with the pathological diagnosis.

Bugs in the system were identified and compared against the first version of the Functional Design (FD). Following this, the analysis phase saw the FD being updated with necessary feedback and remarks. In the design phase, the FD served as the foundation for submitting changes for modeling in the LogicNets© Designer. The implementation phase involved translating the requirements into code and transforming them into software programs. The testing phase commenced after the modeling of changes in the LogicNets© Designer.

The deployment phase marked the creation of a new test link for the SSR tool. Subsequently, in the review phase, the SSR tool’s test links were sent out for evaluation to assess the behavior and validity of the new version. If any errors were found, the process would start new from the requirement-gathering phase. Finally, the maintenance phase addressed any bugs, errors, or the need for updates after the SSR tool’s deployment in the working environment, involving debugging and the addition of new features.

To enhance the tool’s validation, we referred to the development process of the national placenta protocol of Palga (the national pathology databank in the Netherlands), which also utilized iterative rounds of expert feedback to refine the protocol. This approach underscores the robustness of our validation process and aligns with established methods in the field. All calculations were validated through comparison with the national Palga Placenta protocol used in clinical practice [22].

The project completed four full cycles of iterations, emphasizing a methodical and thorough approach to developing and refining the SSR tool to ensure it met the high standards required for pathology data reporting [23].

## Results

### Placenta protocol

The SSR tool for the recording of placental lesions integrates features for the assessment of placental pathology, structured around several key tabs (Demographics, Macroscopy, Microscopy, and Group and Phenotype), each designed to capture specific data fields.

The Demographics tab accommodates patient information, including optional entries such as date of birth and study code, while facilitating linkage to additional protocols through accession numbers. This functionality is vital for keeping a continuous record for patients in instances of multiple births and includes an option to start a distinct case for situations with triplets and quadruplets. A demo version of the SSR Tool is available online (https://ppp-dev.logicnets.net/ppp/demo).

**Fig. 2:**
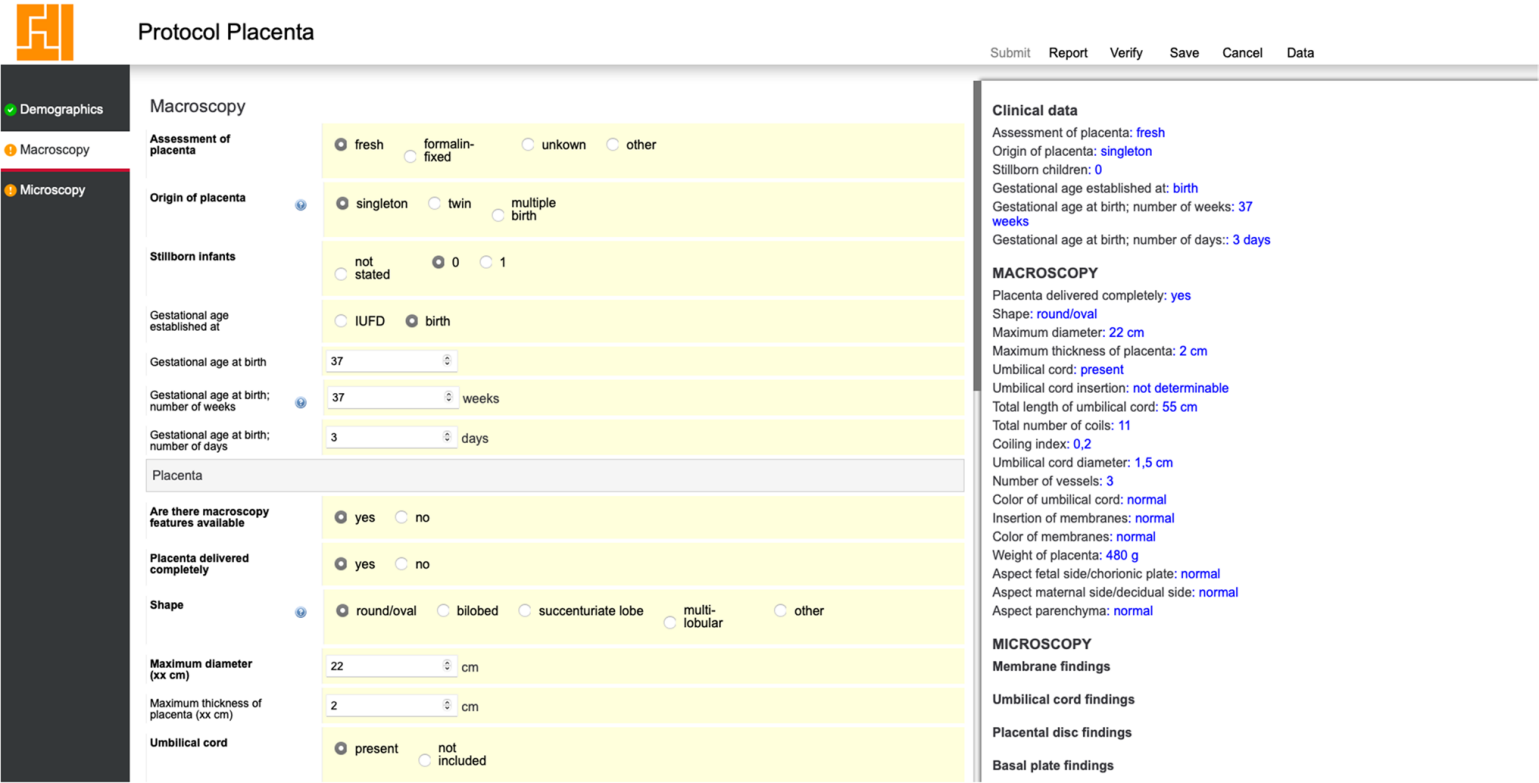
Screenshot of the tool with core (bold) and non-core macroscopic items

The SSR tool mandates the inclusion of data on macroscopic (gross) placental evaluations (Figure 2). A formal uniform reporting method is proposed to replace the current dependence on unstructured text, promoting uniform documentation of macroscopic observations. This approach is in line with the guidelines from the 2016 Amsterdam Placental Workshop Group Consensus Statement, encompassing assessments of the placenta, the number of fetuses (single, twins, or multiples), the status of the infants (liveborn or stillborn), and the gestational age. Attributes such as the placenta’s macroscopic appearance, completeness of the placenta, placental shape, and the characteristics of the umbilical cord are evaluated and recorded. The tool automates calculations, such as the coiling index and the comparison of umbilical cord diameter to gestational age within established normal ranges (Figure 2). This automation enhances efficiency and reduces the need for supplementary tools. Placental weight is recorded, noting whether the placenta was trimmed before weighing (as mentioned in the information box at the question mark), and provides automatic calculation of deviation from expected weight percentiles based on gestational age. In cases involving multiple pregnancies, the tool incorporates inquiries about the septum, estimated placental share, and vascular anastomoses, including connections between twins.

**Fig. 3:**
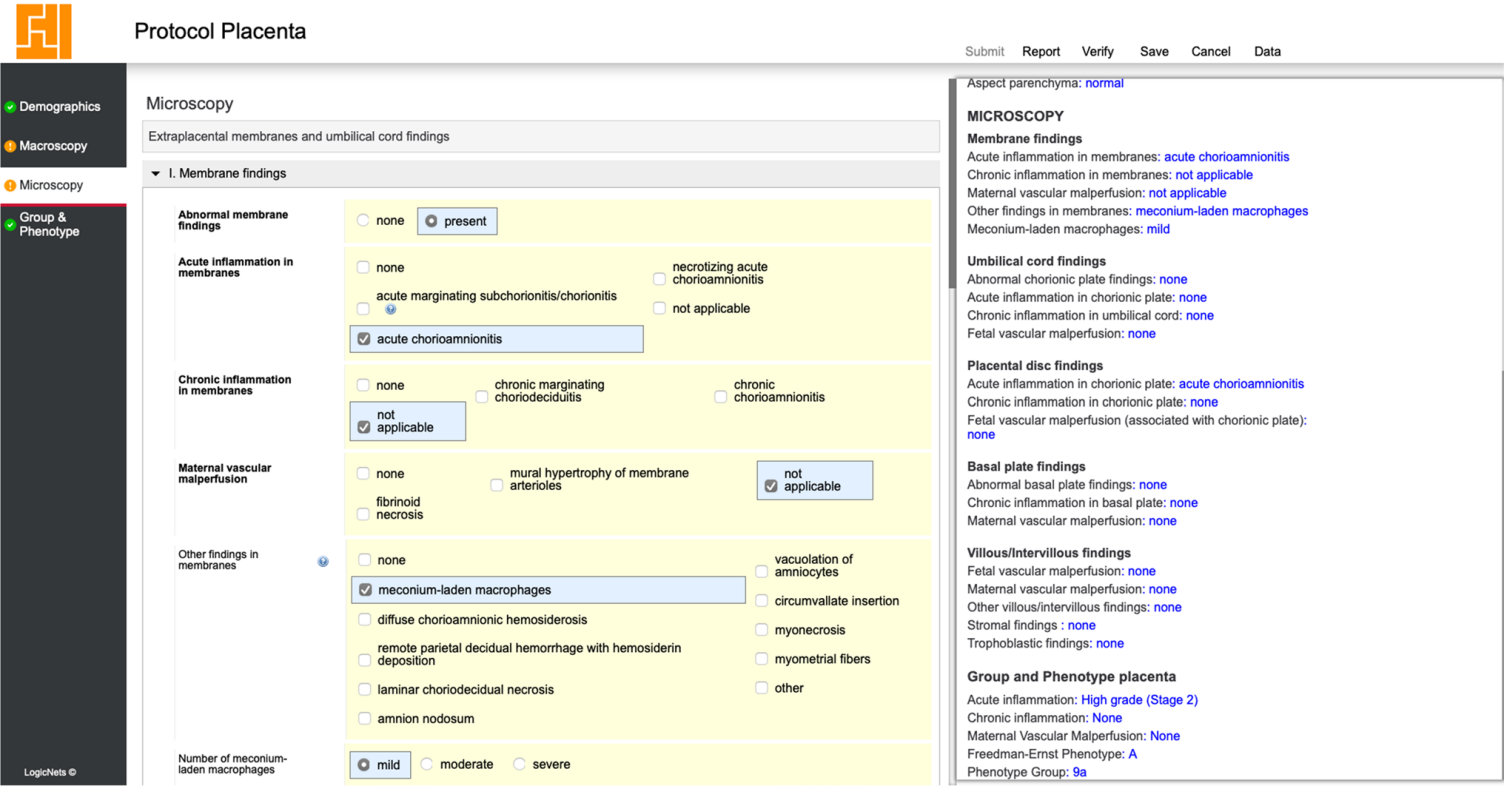
Screenshot of the tool with core (bold) and non-core macroscopic items

The SSR tool’s microscopic assessment component adopts the Freedman-Ernst placental phenotype classification system, modified to align pathology with anatomical sites. Placental lesions, initially categorized into acute inflammation (AI), chronic inflammation (CI), fetal vascular malperfusion (FVM), and maternal vascular malperfusion (MVM), are graded and linked to specific anatomical locations (Table 1). Such a structured approach not only adheres to the Amsterdam Criteria for placental examination but also introduces the flexibility needed to capture a wide array of microscopic details. The Microscopy tab in the SSR tool provides an expansive microscopic examination, covering both extra-placental disc areas, such as the membranes and the umbilical cord, and placental disc components, like the chorionic plate, basal plate, villous/intervillous space, as well as additional placental findings (Figure 3).

**Table 1:** Summary of pathology by pattern of injury (using Freedman-Ernst/Amsterdam Grading)

**Variable**	**Lesions included**	**Grade**	**Definitions**
**Acute inflammation**	*Membrane findings:* acute marginating subchorionitis/chorionitis; acute chorioamnionitis; necrotizing acute chorioamnionitis *Umbilical cord findings:* acute umbilical phlebitis; acute umbilical arteritis; acute funisitis; acute necrotizing funisitis; acute peripheral funisitis *Chorionic plate findings:* acute subchorionitis; acute chorionitis; acute chorioamnionitis; acute necrotizing chorioamnionitis; acute chorionic vasculitis *Villous/Intervillous findings*: acute villitis/intervillositis	[High grade/low grade/none] Abbreviations: A - high-grade acute inflammation a - low-grade acute inflammation	High grade: high-stage maternal and/or fetal^a^, based on Amsterdam Criteria Low grade: low-stage maternal and/or fetal^a^, based on Amsterdam Criteria None: no acute inflammatory lesions
**Chronic inflammation**	*Membrane findings*: chronic marginating choriodeciduitis; chronic chorioamnionitis *Umbilical cord findings*: eosinophilic T-cell vasculitis *Chorionic plate findings*: chronic chorionitis; eosinophilic T-cell vasculitis *Basal plate findings*: chronic deciduitis (with/without plasma cells); chronic decidual perivasculitis; chronic basal villitis *Villous/intervillous findings:* chronic villitis; chronic histiocytic intervillositis	[High grade/low grade/none] Abbreviations: C - high-grade chronic inflammation c - low-grade chronic inflammation	HG - ≥2 compartments with chronic inflammation (C) LG - 1 compartment with chronic inflammation (c) None - 0 compartments with chronic inflammation (no chronic inflammatory lesions)
**Fetal vascular malperfusion**	*Umbilical cord findings:* umbilical vein thrombus/intramural fibrin deposition; umbilical artery thrombus/intramural fibrin deposition *Chorionic plate findings:* fetal vascular thrombi/intramural fibrin deposition; large chorionic vessel(s) abnormalities *Villous/intervillous findings:* thrombi/intramural fibrin deposition in small stem vessels; avascular villi; villous stromal-vascular karyorrhexis	[High grade/low grade/none] Abbreviations: F - high-grade fetal vascular malperfusion f - low-grade fetal vascular malperfusion	High grade: High-grade FVM or fetal thrombotic vasculopathy, defined as more than one focus of avascular villi (45 villi) or two or more occlusive or nonocclusive thrombi in the chorionic plate or stem villi, or multiple nonocclusive thrombi Low grade: any fetal vascular lesions not indicated as high grade None: no fetal vascular lesions
**Maternal vascular malperfusion:**	*Membrane findings*: mural hypertrophy of membrane arterioles; fibrinoid necrosis of membrane arterioles *Basal plate findings*: muscularization of basal plate arterioles; fibrinoid necrosis/acute atherosis; basal decidual vascular thrombus; retroplacental blood/hematoma; retroplacental hematoma with hemosiderin or infarct (infarction with hemorrhage) *Villous/intervillous findings:* villous infarct(s) – single or multiple; accelerated villous maturation (increased syncytial knots); distal villous hypoplasia; villous agglutination	[High grade/low grade/none] Abbreviations: M - high-grade maternal vascular malperfusion m - low-grade maternal vascular malperfusion	High grade: Score of ≥4 (M) Low grade: Score of 2–3 (m) None: score of 0–1 1 point: fibrinoid necrosis/acute atherosis, muscularization of basal plate arterioles, mural hypertrophy of membrane arterioles, basal decidual vascular thrombus, single infarct, increased syncytial knots, villous agglutination, increased perivillous fibrin deposition, distal villous hypoplasia, retroplacental blood/hematoma 2 points: multiple infarcts, retroplacental hematoma with hemosiderin or infarct (infarction with hemorrhage), low placental weight/placental hypoplasia^b^
Other significant pathology	*Membrane findings*: meconium-laden macrophages, diffuse chorioamnionic hemosiderosis, remote parietal decidual hemorrhage with hemosiderin deposition, laminar choriodecidual necrosis, amnion nodosum, vacuolation of amniocytes, circumvallate insertion with/without marginal hemorrhage, myonecrosis, and myometrial fibers *Chorionic plate findings*: chorion nodosum, myonecrosis, diffuse chorioamnionic hemosiderosis, focal chorionic hemosiderin deposition, meconium-laden macrophages *Basal plate findings*: acute intradecidual hemorrhage, focally disrupted maternal surface with fresh hemorrhage, basal plate hemosiderin deposition, basal plate myometrial fibers with decidua, and meconium-laden macrophages *Villous/intervillous findings:* increased/massive perivillous fibrin deposition, maternal floor infarct, intervillous thrombus, extravillous trophoblast cyst(s), massive subchorial hematoma, placental mesenchymal dysplasia, delayed villous maturation, intervillous space infiltrates (intraplacental choriocarcinoma, intervillous metastatic carcinoma, intervillous sickling/pigment/organisms, intravillous viral inclusion) *Other placental disc findings (additional):* *1. Stromal findings:* villous edema (diffuse/focal or pathy), extramedullary hemopoiesis, intravillous acute hemorrhage 2. *Trophoblastic findings*: persistent cytotrophoblast, trophoblast or stromal vacuolation (in trophoblastic cell, stromal Hofbauer cell, villous stromal fibroblast, endothelial cell, amniotic cell), trophoblast stromal inclusions, basement membrane mineralization (calcium deposits/ferric iron) 3. *Vascular findings*: hypervascularity (villous chorangiosis, multifocal chorangiomatosis, chorangioma (sporadic, Multiple Chorangioma Syndrome), increased nucleated erythrocytes in fetal vasculature, intravascular fetal atypical cells/malignancy, fetal vascular involutional changes consistent with fetal demise	Not graded	Presence of ≥ 1 lesion h = amnion nodosum i = myonecrosis j = diffuse chorioamnionic hemosiderosis k = basal plate myometrial fibers with decidua is absent (stage 1) s = basal plate myometrial fibers with decidua is present (stage 2) n = increased perivillous fibrin deposition o = massive perivillous fibrinoid deposition p = delayed villous maturation q = villous chorangiosis r = multifocal chorangiomatosis

^a^The overall acute inflammation grade reflects combined maternal and/or fetal responses, based on Amsterdam Criteria. Individual components (maternal/fetal stages) are captured discretely in the tool and can be independently analyzed or categorized in future classification models.

^b^Low placental weight/placental hypoplasia (placental trimmed weight < 10^th^ percentile that is incorporated into the tool [24]) is only considered as MVM in the presence of at least another maternal vascular lesion (isolated placental hypoplasia is not scored)

A distinctive feature of the SSR Tool is its ability to synthesize data collected across both macroscopic and microscopic examinations to automatically generate coded phenotype groups and assign Freedman-Ernst phenotypes (Table 2). This automation enhances the standardization of findings and coding pathologies, further refining the specificity and standardization of the diagnostic output.

**Table 2:** Summary of pathology by group according to Freedman-Ernst

**Group name and description**	**Group number**	**Included phenotypes**
**Multiple high-grade pathologies (C, F, M)**	1	
All three high-grade pathologies (triple threat)	1a	CFM, aCFM, ACFM
High-grade fetal AND maternal vascular pathology	1b	FM, cFM, aFM, acFM, AFM, AcFM
High-grade maternal vascular pathology AND high-grade chronic inflammation	1c	CM, CfM, aCM, aCfM, ACM, ACfM
High-grade fetal vascular pathology AND high-grade chronic inflammation	1d	CF, CFm, aCF, aCFm
**High-grade acute inflammation AND high-grade fetal vascular pathology (with or without chronic inflammation and/or low-grade maternal vascular pathology)**	2	AF, AFm, AcF, AcFm, ACF, ACFm
**High-grade fetal vascular pathology**		
With or without low-grade acute inflammation	3a	F, aF
WITH low-grade chronic inflammation AND/OR low-grade maternal vascular pathology (with or without low-grade acute inflammation)	3b	cF, Fm, cFm, acF, aFm,acFm
**High-grade chronic inflammation**		
With or without low-grade fetal vascular pathology	4a	C, Cf
WITH any acute inflammation (with or without low-grade fetal vascular pathology)	4b	aC, aCf, AC, Acf
WITH low-grade maternal vascular pathology (with or without low-grade fetal vascular pathology)	4c	Cm, Cfm
WITH low-grade maternal vascular pathology AND any acute inflammation	4d	aCm, aCfm, ACm, Acfm
**High-grade maternal vascular pathology (with or without any acute inflammation and/or other low-grade pathology)**	5	M, cM, fM, cfM, aM, acM, afM, acfM, AM, AcM, AfM, AcfM
**Low-grade fetal vascular pathology OR chronic inflammation**		
With or without low-grade acute inflammation	6a	c, f, ac, af
WITH high-grade acute inflammation	6b	Ac, Af
**Low-grade maternal vascular pathology OR any combination of low-grade chronic inflammation and fetal vascular malperfusion**		
Without acute inflammation	7a	m, cm, cf, fm, cfm
WITH low-grade acute inflammation	7b	am, acm, acf, afm, acfm
WITH high-grade acute inflammation	7c	Am, Acm, Acf, Afm, Acfm
**Other significant pathology**	8	h, i, j, k, s, n, o, p, q, r
**Acute inflammation alone**		**Only acute inflammation (either high or low grade)**
High-grade acute inflammation	9a	A
Low-grade acute inflammation	9b	a
**Histologically normal**	10	“None” in all the findings

## Discussion

### Principal findings

The placenta SSR tool is developed to provide uniform reporting and automated diagnostic category assignments at various levels of detail. By incorporating both core and non-core items, the tool allows pathologists to control the granularity of their reporting. It achieves Level 5 structured reporting according to the Ontario project’s categorization, using standardized, discrete data elements presented in templates similar to those offered by the ICCR [25]. This level enables significant advancements in electronic data capture through user-friendly interfaces such as radio buttons, checkboxes, and drop-down menus. The main findings confirm the tool’s effectiveness in improving data quality and ensuring regional interoperability. However, while level 5 structured reporting enhances data integration with clinical records and ensures regional consistency, it does not involve terminology binding—a feature exclusive to level 6 and necessary for achieving full standardization on a global scale. This balance of significant improvements and acknowledged limitations highlights the tool’s potential without overstating its current capabilities.

#### Results in the context of what is known

The SSR tool for placental examination substantially improves the consistency, completeness, and precision of pathology reports compared to narrative pathology reports, enhancing both clinical and research applications of placental pathology reports [26, 27]. These improvements are consistent with current trends in medical research that emphasize the development of digital diagnostic tools [21]. Unlike other systems, the SSR tool introduces a higher level of structured reporting that is rarely observed in the field of placental pathology. It uniquely combines essential and additional elements, facilitating comprehensive yet adaptable reporting and distinguishing itself from existing tools that either lack such detailed features or do not allow for customizable data entry.

The SSR tool addresses several current limitations by incorporating structured and standardized mechanisms for macroscopic and microscopic assessments. Incorporating built-in registration controls is a key aspect of the SSR tool, offering a blend of core and non-core questions that promote a uniform and methodical reporting process. This framework aims to enhance the accuracy, uniformity, and completeness of reported data while also offering users the flexibility to control the level of detail. However, it is important to note that we do not provide direct comparative data to substantiate improvements in data quality over previous methods. While the SSR tool enhances data collection in settings where general pathologists operate or resources are scarce, interpretation of findings is still reliant on the expertise of pathologists.

The SSR tool enhances its utility through features like analysis capabilities for multiple births, advanced security measures, and seamless database integration, making it applicable across many research projects. It is designed to improve reporting accuracy and standardization, facilitating clear communication among healthcare professionals. With strict security protocols, the tool ensures the protection of sensitive health information, adhering to privacy and ethical guidelines. The tool is designed with durability and flexibility in mind, allowing for future integration of new findings in placental pathology. However, such updates must be approached with caution, as changes to criteria, terminology, or database structure can compromise longitudinal data comparability, particularly when new definitions or categories disrupt consistency with earlier datasets. This challenge has been seen in other fields, such as the shift from the 7th to 8th editions of the TNM classification in oncology. To address this, rigorous version control and change documentation will be essential in future SSR tool iterations. Thanks to its modular design, with separate scoring of histological features, the tool can be adapted relatively easily if definitions evolve in future updates to the Amsterdam criteria. An essential characteristic of the SSR tool is its comprehensive database integration, which is crucial for research by enabling efficient data storage and access while ensuring data security. This feature is particularly beneficial for large-scale studies supporting research in placental pathology. Promoting the widespread use of such standardized tools is essential for achieving consistency and reproducibility in placental examinations, ultimately improving patient outcomes and expanding scientific knowledge.

### Clinical implications

The design and functionality of the SSR tool indicate it has the potential to enhance the efficiency of placental examinations, leading to quicker and more accurate diagnoses. These improvements could influence clinical outcomes by providing pathologists and researchers with more reliable data faster. However, the broader clinical adoption of the tool faces several hurdles. The SSR tool was initially designed exclusively for research applications and is currently employed in the placenta analysis of various research studies, notably “DRIGITAT” (Doppler Ratio In fetal Growth Restriction Intervention Trial At Term) and “CEPRA” (The Cerebro Placental Ratio as an indicator for birth at reduced fetal movements) [28–31]. In future studies, a conclusion will be included in the SSR, gathering clinician feedback and evaluating the tool’s effectiveness in clinical practice.

Additionally, the tool’s complexity and outputs may require training and adaptation to ensure clinicians’ usability. Simplifying the presentation of data and including interpretative comments may enhance its utility in clinical settings. The regulatory landscape adds complexity, as clinical use in the EU requires compliance with specific regulations and CE marking for medical devices. This demands careful navigation of norms, laws, and regulations, further complicating clinical integration.

### Research implications

Several questions remain unanswered about the broader application of the SSR tool, particularly its adaptability and effectiveness in diverse settings. Future research should focus on evaluating the long-term impacts of using such a tool in both clinical and research environments. This includes conducting longitudinal studies to track its reliability and effectiveness over time, and comparative effectiveness research to measure it against other diagnostic tools. Proposals for future studies should also consider implementation research to identify and overcome barriers to adoption and usability studies to refine the tool based on user feedback from pathologists and clinical staff. Randomized controlled trials to assess the impact on clinical outcomes and qualitative studies to gather detailed user experiences will enhance our understanding of the tool’s practical benefits and limitations. Engaging with clinicians in the validation process will be crucial to ensure the tool meets the needs of all stakeholders.

It is also important to note that the SSR tool was developed within a research setting and is optimized for use in term and third-trimester placentas. Although some components may apply to second-trimester specimens, its use in early gestation, particularly in first-trimester placentas, is currently unsupported due to the lack of validation and the distinct histological features of early placental development. Future research should explore the feasibility and accuracy of extending the tool to earlier gestational ages.

### Strengths and limitations

While the SSR tool has the aforementioned strengths, such as providing consistent, structured level 5 reporting and integrating seamlessly with existing clinical data systems, setting it apart from many contemporary diagnostic tools, it also faces several limitations restricting its wider use. The significant financial investment required for its development, maintenance, and updates presents a substantial barrier, potentially deterring resource-limited institutions from adopting this technology, particularly in clinical environments. The tool features a high level of detail, including some non-core elements, requiring pathologists to invest time and effort to adapt to its use.

The SSR tool has the potential for significant evolution, and future iterations should aim for deeper integration with other established medical terminologies, such as SNOMED CT. Advancing from level 5 to level 6 in terminology integration would improve the tool’s precision, standardization, and compatibility with broader healthcare systems. Such enhancement facilitates effective communication among medical professionals.

The long-term vision for the SSR tool is to transform it into a fully integrated clinical tool. This evolution requires the development of robust mechanisms for conclusion integration, continuous updates, and maintenance within clinical environments. Addressing these challenges involves not only financial investment but also collaborative efforts among developers, medical professionals, and regulatory bodies. Devising effective cost management and funding strategies could alleviate financial barriers, enabling continuous improvement and updates necessary for the tool’s relevance and effectiveness.

To ensure the SSR tool remains compatible and functional across different systems, it is critical that updates are grounded in solid evidence and conform to both national and international guidelines. Ensuring that vendors meet these standards is crucial, as is the push towards a more modular approach. Keeping in step with technical norms and fostering a culture of interoperability are fundamental aspects. Moreover, instituting rigorous testing, providing comprehensive training, and establishing a collaborative governance structure is vital, along with ensuring that incentives, regulatory measures, and financial backing are properly aligned [32]. Continual research and development efforts are essential to improve the SSR tool’s performance, enhance precision, and make it more user-friendly. Engaging with the wider medical and research communities is key to gathering valuable feedback and insights, which will help in the ongoing development and refinement of the tool. Widespread adoption and its consistent application across different institutions are crucial for standardizing placental pathology reporting, which, in turn, can significantly improve the quality of research and contribute to better clinical outcomes.

## Conclusion

The creation of the SSR tool represents progress in standardizing research reporting of placental pathology, aiming to bridge the gap between current challenges and future enhanced diagnostic precision. It supports an ambition to foster clearer communication among healthcare professionals and improve outcomes for mothers and infants. While the tool currently focuses on standardizing reporting among pathologists (researchers), future efforts will include clinician feedback and validation to enhance its clinical utility.
